# Measurement of Visceral Fat: Should We Include Retroperitoneal Fat?

**DOI:** 10.1371/journal.pone.0112355

**Published:** 2014-11-17

**Authors:** Chi-Sheng Hung, Jen-Kuang Lee, Chung-Yi Yang, Hung-Ren Hsieh, Wen-Ya Ma, Mao-Shin Lin, Pi-Hua Liu, Shyang-Rong Shih, Jyh-Ming Liou, Lee-Ming Chuang, Ming-Fong Chen, Jou-Wei Lin, Jung-Nan Wei, Hung-Yuan Li

**Affiliations:** 1 Department of Internal Medicine, National Taiwan University Hospital, Taipei, Taiwan; 2 Department of Clinical Pathology, Far Eastern Memorial Hospital, New Taipei City, Taiwan; 3 Department of Medical Imaging, National Taiwan University Hospital and National Taiwan University College of Medicine, Taipei, Taiwan; 4 Department of Radiology, National Taiwan University Hospital Yun-Lin Branch, Yun-Lin, Taiwan; 5 Division of Endocrinology, Department of Internal Medicine, Cardinal Tien Hospital, Xindian, Taiwan; 6 Clinical Informatics and Medical Statistics Research Center, Chang Gung University, Gueishan, Taiwan; 7 Department of Internal Medicine, National Taiwan University Hospital Yun-Lin Branch, Yun-Lin, Taiwan; 8 Chia Nan University of Pharmacy and Science, Tainan, Taiwan; University of Leicester, United Kingdom

## Abstract

**Objective:**

Whether retroperitoneal fat should be included in the measurement of visceral fat remains controversial. We compared the relationships of fat areas in peritoneal, retroperitoneal, and subcutaneous compartments to metabolic syndrome, adipokines, and incident hypertension and diabetes.

**Methods:**

We enrolled 432 adult participants (153 men and 279 women) in a community-based cohort study. Computed tomography at the umbilicus level was used to measure the fat areas.

**Results:**

Retroperitoneal fat correlated significantly with metabolic syndrome (adjusted odds ratio (OR), 5.651, p<0.05) and the number of metabolic abnormalities (p<0.05). Retroperitoneal fat area was significantly associated with blood pressure, plasma glycemic indices, lipid profile, C-reactive protein, adiponectin (r = −0.244, P<0.05), and leptin (r = 0.323, p<0.05), but not plasma renin or aldosterone concentrations. During the 2.94±0.84 years of follow-up, 32 participants developed incident hypertension. Retroperitoneal fat area (hazard ration (HR) 1.62, p = 0.003) and peritoneal fat area (HR 1.62, p = 0.009), but not subcutaneous fat area (p = 0.14) were associated with incident hypertension. Neither retroperitoneal fat area, peritoneal fat area, nor subcutaneous fat areas was associated with incident diabetes after adjustment.

**Conclusions:**

Retroperitoneal fat is similar to peritoneal fat, but differs from subcutaneous fat, in terms of its relationship with metabolic syndrome and incident hypertension. Retroperitoneal fat area should be included in the measurement of visceral fat for cardio-metabolic studies in human.

## Introduction

Metabolic syndrome (MS) is a combination of risk factors mainly related to abdominal obesity and insulin resistance, according to the National Cholesterol Education Program’s Adult Treatment Panel III report (ATP III) [1]. Participants with MS have a higher risk of fatal and nonfatal cardiovascular diseases [2]. Abdominal or central obesity plays an important role in the development of MS. Indeed, central obesity is a requirement to meet the criteria of MS as defined by the International Diabetes Federation [3]. Abdominal adipose tissue is not only a fat storage site, but also acts as an endocrine organ that secretes various adipokines such as adiponectin and leptin as well as several inflammatory cytokines. In addition, the free fatty acid flux from abdominal adipose tissue to the liver results in disturbances in glucose and lipid metabolism [4], which contribute to the development of insulin resistance and dyslipidemia in participants with MS [5]. [6].

In the abdominal cavity, there are 3 different compartments of fat: omental, mesenteric, and retroperitoneal fat. Blood from fat tissue in the peritoneal region, including omental and mesenteric fat, is drained through the portal vein into the liver, while the blood from fat in the retroperitoneal region is drained into the kidney, pancreas, or directly to the vena cava [7]. Furthermore, the composition and amount of adipokines released from different compartments can differ, and along with their endocrine effects, these adipokines can have paracrine effects on adjacent organs. Therefore, peritoneal and retroperitoneal fats may play different roles in our metabolism. Two recent reports in animals have shown that peritoneal and retroperitoneal fat have different immuno-modulatory roles and respond differently to exercise training [8], [9]. However, most human studies using CT or MRI to assess the degree of visceral obesity have included the retroperitoneal fat area [10]–[12]. In the literature, retroperitoneal and peritoneal fat depots were quantified together as visceral fat, to study their relationships to cardiometabolic diseases. To the best of our knowledge, there is no report investigating the role of retroperitoneal fat, independent to other part of visceral fat, in metabolic abnormalities in humans. Therefore, we conducted this study to compare the relationships of retroperitoneal fat area, peritoneal fat area, and subcutaneous fat area to metabolic syndrome, adipocytokines, and incident hypertension and diabetes. Patterns of the relationships can help us to answer if we should include retroperitoneal fat when measuring visceral fat area in humans.

## Methods and Materials

### Ethic statement

The study was approved by the Institutional Review Board of the National Taiwan University Hospital, and complied with the Helsinki Declaration. Written informed consent was obtained from each patient before enrollment.

### Participants

From 2006 to 2012, residents from the Yunlin county, Taiwan, aged ≥18 years, who did not report the presence of diabetes during an interview, were invited to join this prospective study, named the Taiwan Lifestyle Study [13], [14]. There were 3 visits for this prospective study, separated by 1–3 years. Individuals underwent abdominal CT exam at the 2nd visit, which was defined as the baseline visit in the present study. The 3^rd^ visit was defined as the follow-up visit. The new diabetes or hypertension was defined by comparing the status between baseline and follow-up visit. Written informed consent was obtained from every participant. The study was review and approved by the Institutional Review Board.

A questionnaire was administered by trained nurses in order to obtain data on the demographic characteristics, medical history, and health habits of the participants. Body height and weight were recorded to the nearest 0.5 cm and 0.1 kg, respectively. BMI was calculated from body weight in kilogram divided by the square of body height in meters. Waist circumferences were measured according to the method by the World Health Organization and the International Diabetes Federation to the nearest 0.1 cm [15]. Blood pressure was recorded using a mercury sphygmomanometer to the nearest 2 mmHg with the arm supported at the heart level after the subject sat calmly for 10 min; trained nurses took 3 separate readings at 1-min intervals, and the average of the second and the third readings was used for analysis. A standard 75-g oral glucose tolerance test (OGTT) was performed after fasting overnight for 8 h. All study participants were contacted by telephone, e-mail, or postal mail every 1 to 3 years after the initial visit, and follow-up visits were scheduled according to the respondent’s availability. Abdominal computer tomography was done at baseline visit to measure abdominal fat. Clinical questionnaires, physical examination, and blood tests including OGTT were repeated to know the development of incident hypertension or incident diabetes at the time of follow-up.

Plasma glucose and fasting serum total cholesterol, triglycerides, high-density lipoprotein cholesterol, and low-density lipoprotein cholesterol concentrations were measured using an automatic analyzer (Toshiba TBA 200 FR; Toshiba Medical Systems Co., Ltd., Tokyo, Japan). Plasma hemoglobin A1c (HbA1c) concentrations were measured using another automatic analyzer (HLC-723 G7 HPLC systems; Tosoh Corporation, Tokyo, Japan). Hepatitis B surface antigen and hepatitis C virus antibody were measured by the AxSYM System (Abbott Laboratories, North Chicago, IL). The laboratory attends and is qualified by an external quality assurance program by the Taiwan Society of Laboratory Medicine twice a year. The HbA1c assay was certified by the National Glycohemoglobin Standardization Program [16] and standardized to the Diabetes Control and Complications Trial reference assay.

### Definitions

Diabetes was diagnosed according to the American Diabetes Association (ADA) recommendations in 2010 (HbA1c concentration ≥6.5% (48 mmol/mol), fasting plasma glucose (FPG) concentration ≥126 mg/dL, or OGTT 2h plasma glucose (OGTT-2h-PG) concentration ≥200 mg/dL). MS was diagnosed using the criteria defined in the ATP III, with a modification of waist circumference for Asians [17]. Participants were classified as having MS if they met 3 or more of the following 5 criteria: (1) high blood pressure: systolic and/or diastolic blood pressure ≥130/85 mmHg or receiving blood pressure-lowering medications; (2) hyperglycemia: fasting plasma glucose concentration ≥100 mg/dL (5.6 mmol/L) or receiving glucose-lowering medications; (3) hypertriglyceridemia: fasting plasma triglyceride concentration ≥150 mg/dL (1.69 mmol/L); (4) HDL cholesterol concentration <40 mg/dL in men and <50 mg/dL in woman; and (5) waist circumference ≥90 cm in men and ≥80 cm in women.

### Measurements of abdominal adipose tissue

**Figure 1:**
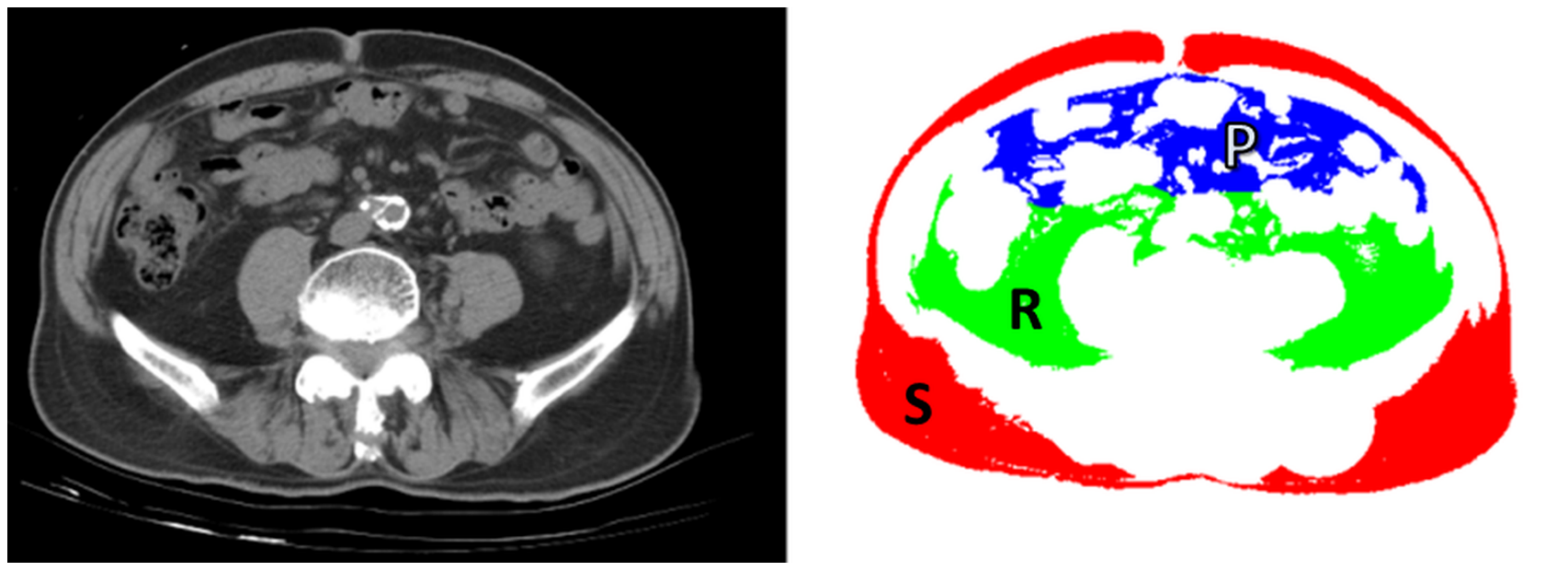
Image demonstration of determining abdominal fat distribution on a CT scan. Left, sample CT image obtained at the umbilicus level. Right, fat masks created for determining areas of subcutaneous fat (red, “S”), peritoneal fat (blue, “P”) and retroperitoneal fat (green, “R”) using methods described in the Materials and Methods section.

Abdominal adiposity was assessed using a 16-slice multidetector CT scanner (LightSpeed 16; GE Healthcare, Milwaukee, WI) in the supine position (120 kVp, 400 mAs, slice thickness of 5 mm). Image analysis software (ImageJ, version 1.44; National Institutes of Health, Bethesda, MD) was used to quantify the subcutaneous and visceral adipose fat areas, on one cross-sectional scan obtained at the level of umbilicus and expressed in millimeters squared. [11] After applying threshold with an attenuation range of –50 to –250 Hounsfield units, a fat-density mask was generated. A total fat mask was created after manual exclusion of non-adipose area (such as the CT table, air-object interface or fecal material) from the fat-density mask (Figure 1). The visceral fat area was determined by cutting areas other than visceral fat, and the subcutaneous fat area was calculated with the total fat area subtracted by the visceral fat area. The visceral fat area was further divided into peritoneal and retroperitoneal areas along the boundary comprised of posterior surface of small bowel, ascending colon, descending colon, mesenteric vessels and gonadal veins (Figure 1). The interfascial plane could be visualized as a thin line in some participants and this could help outline of the boundary.

### Statistical analysis

The sample size estimation was based on the following assumptions that was modified from a previous study(18): a 2-sided α level of 0.05 and power of 95% and retroperitoneal fat area of 5000 mm^2^ in MS group and 4000 mm^2^ in non-MS group with a SD of 2500 mm^2^. The MS to non-MS group ratio was set at 1∶3 according to a prevalence of MS at around 25–30%. Accordingly, a sample size of 400 patients (100 patients in MS group and 300 patients in non-MS group) was calculated.

All variables are expressed as the mean and standard deviation (SD). Two-sample *t*-tests and Pearson’s chi-square tests were used to compare the demographic and metabolic parameters between participants with and without MS. The different fat areas were logarithmically transformed to approximate normal distributions. Logistic regression models were used to evaluate the relationship between MS and different fat areas using age, gender, and BMI as potential confounders. We also performed this analysis with standardized fat areas as follows: standardized fat area = (log fat area – mean of log fat area)/(SD of log fat area). Pearson’s correlation coefficients were used to assess the relationship between metabolic parameters and different fat areas. Partial correlation coefficients were calculated after adjusting for age and gender. Bootstrap resampling was used to compare the correlation coefficient of each metabolic variable and the retroperitoneal fat area with the correlation coefficient of each metabolic variable and the subcutaneous or peritoneal fat area. The differences in numbers of metabolic abnormalities were assessed by analysis of variance. The relationship between adipokines and the retroperitoneal fat area was expressed by Pearson’s correlation coefficients. Cox proportional hazard models were used to assess the relationship of abdominal fat areas and the development of incident hypertension or incident diabetes during follow-up, adjusted for age, sex, and family history of hypertension or diabetes. The fat areas in the Cox regression models were normalized by the standard deviation, to show the hazard ratios for every 1 standard deviation increase in fat areas. Kaplan-Meier failure curves were used to estimate the cumulative incidence of hypertension and diabetes in participants with fat areas above and below the median value. A two-tailed p value <0.05 was regarded as significant. Statistical analyses were performed with Stata/SE 11.0 for Windows (StataCorp LP, College Station, TX).

## Results

A total of 432 participants (153 men and 279 women), with a mean age of 52.6±12.0 years, were enrolled in this study. Of the 432, 125 participants fulfilled the criteria for MS. The mean fat areas in men and women were as follows: 14,757 mm^2^ and 19,659 mm^2^, respectively, of subcutaneous fat area (p<0.001); 7,187 mm^2^ and 4,855 mm^2^, respectively, of peritoneal fat area (p<0.001); and 5,233 mm^2^ and 3,784 mm^2^, respectively, of retroperitoneal fat area (p<0.001). As shown in Table 1, participants with MS were older; more obese; more likely to have hypertension and diabetes; had higher plasma triglyceride, glutamyl pyruvic transaminase (GPT), leptin, and C-reactive protein (CRP) concentrations, had lower plasma adiponectin concentrations, and had higher subcutaneous, peritoneal, and retroperitoneal fat areas.

**Table 1:** Clinical characteristics of participants with and without metabolic syndrome (MS).

	No MS	MS	*p* value
N	307	125	
Age (years)	50.8 (12.2)	57.1 (10.2)	<0.001
Gender (male (%))	97 (31)	56 (44.8)	<0.001
Systolic blood pressure (mmHg)	121 (16)	133 (15)	<0.001
Diastolic blood pressure (mmHg)	77 (9)	83 (10)	<0.001
Medications for hypertension (n, %)	24 (7.8)	37 (29.6)	<0.001
Hypertension (n, %)	68 (22.1)	68 (54.4)	<0.001
Body mass index (kg/m^2^)	23.1 (3.0)	26.4 (2.9)	<0.001
Waist circumference (cm)	80.4 (7.3)	89.8 (7.1)	<0.001
Fasting plasma glucose (mg/dL)	91.2 (10.2)	102.2 (21.2)	<0.001
OGTT-2h-PG (mg/dL)	116.5 (36.2)	158.9 (71)	<0.001
Hemoglobin A1c (%)	5.7 (0.4)	6.0 (1.0)	<0.001
Hemoglobin A1c (mmol/mol)	39(2.7)	42(6.8)	<0.001
HOMA2%B	81.6 (34.9)	87.9 (41.2)	0.1
HOMA2%S	156.8 (72.2)	108.6 (60)	<0.001
Medications for diabetes (n, %)	4 (1.3)	9 (7.2)	0.003
Diabetes (n, %)	26 (8.5)	34 (27.2)	<0.001
Total cholesterol (mg/dL)	193.7 (35.7)	196.4 (36.1)	0.243
Triglyceride (mg/dL) a	93.2 (62–111)	189.3 (113–209)	<0.001
HDL cholesterol (mg/dL)	53.4 (10.5)	42.3 (8.2)	<0.001
LDL cholesterol (mg/dL)	118.7 (31.8)	122.3 (35.8)	0.149
Medications for dyslipidemia (n, %)	4 (1.3)	16 (12.8)	<0.001
Glutamate oxalate transaminase (IU/L)	23.1 (12.4)	23.6 (7.5)	0.35
Glutamate pyruvate transaminase (IU/L)	22.6 (20.7)	27.3 (16.8)	0.012
Leptin (pg/mL) a	9645 (3873–12844)	12093 (5576–15835)	0.002
Adiponectin (ng/mL) a	8040 (3678–10850)	5441 (2489–7647)	<0.001
Renin (pg/mL) a	27.6 (9.3–37.3)	32.3 (10.5–40.7)	0.24
Aldosterone (pg/mL) a	48.5(22.1–61.5)	43.4 (20.3–54.1)	0.47
C-reactive protein (mg/dL) a	0.13 (0.04–0.15)	0.24 (0.06–0.24)	<0.001
Subcutaneous fat (mm^2^) a	16588 (6935)	21203 (6769)	<0.001
Peritoneal fat (mm^2^) a	4610 (3075)	8303 (3448)	<0.001
Retroperitoneal fat (mm^2^) a	3629 (1925)	5927 (2154)	<0.001

Means (standard deviations) are shown.

a Medians (interquartile ranges) of variables not normally distributed are shown. Statistical analyses were performed after log transformation.

OGTT-2h-PG, plasma glucose at 2 h during oral glucose tolerance test; HOMA, homeostasis model assessment; HDL, high-density lipoprotein; LDL, low-density lipoprotein.

### Retroperitoneal fat area is associated with metabolic syndrome

**Figure 2:**
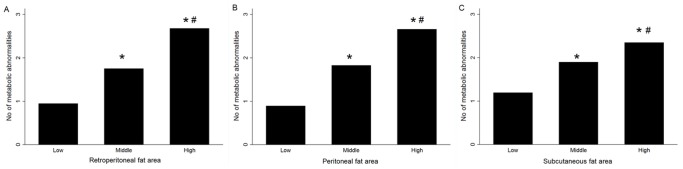
Clustering of metabolic abnormalities defined by MS in participants with body fat areas in the lowest, middle, or highest tertiles. (A) Retroperitoneal fat, (B) peritoneal fat, and (C) subcutaneous fat. *p<0.05 vs. lowest tertile, #p<0.05 vs. middle tertile.

As shown in Figure 2, participants with higher fat areas had more metabolic abnormalities in all compartments of abdominal fat (all p<0.05). In multivariate logistic regression analysis (Table 2), retroperitoneal and peritoneal fat areas, but not the subcutaneous fat area, were significant predictors for the presence of MS, independent of age, gender, and BMI (retroperitoneal fat: OR, 5.651; 95% CI, 2.707–11.79; p<0.05; peritoneal fat: OR, 3.991; 95% CI, 2.181–7.304; p<0.05; subcutaneous fat: OR, 2.569; 95% CI, 0.935–7.053; p>0.05). The odds ratios for every 1 SD increase in fat area, adjusted for age, gender, and BMI, were 1.562 (p>0.05) for the subcutaneous fat area, 3.489 (p<0.05) for the peritoneal fat area, and 2.849 (p<0.05) for the retroperitoneal fat area.

**Table 2:** The relationship between metabolic syndrome and body fat in logistic regression models, using metabolic syndrome as the dependent variable.

	Model 1	Model 2	Model 3
Age	1.045^a^ (1.022–1.068)	1.032^a^ (1.008–1.057)	1.034^a^ (1.009–1.058)
Male gender	1.615 (0.840–3.106)	0.797 (0.471–1.345)	0.79 (0.465–1.343)
Body mass index	1.292^a^ (1.147–1.463)	1.241^a^ (1.121–1.373)	1.245^a^ (1.126–1.377)
Subcutaneous fat	2.569 (0.935–7.053)		
Peritoneal fat		3.991^a^ (2.181–7.304)	
Retroperitoneal fat			5.651^a^ (2.707–11.79)

Body fat was logarithmically transformed for statistical analyses. Odds ratios (95% CI) were shown.

a p<0.05.

### Retroperitoneal fat area is associated with adipocytokines

Of the study population, 353 participants did not take any medication for hypertension, diabetes, or dyslipidemia. In these participants, there were significant correlations between metabolic variables and the retroperitoneal as well as peritoneal fat area (Table 3). The retroperitoneal fat area was significantly associated with blood pressure, BMI, waist circumference, plasma glycemic indices, lipid profile, CRP concentration s, GPT concentrations, leptin concentrations, and adiponectin concentrations, but not with plasma renin and aldosterone concentrations. The associations between retroperitoneal fat area and blood pressure, BMI, waist circumference, fasting glucose, total cholesterol, and LDL cholesterol were independent to peritoneal fat area. The retroperitoneal fat area correlated better with diastolic blood pressure and waist circumference as compare to the peritoneal fat area, whereas the peritoneal fat area correlated better with low HDL cholesterol, triglyceride, glutamyl oxaloacetic transaminase (GOT), and GPT concentrations. In contrast, the subcutaneous fat area was not significantly associated with blood pressure, fasting plasma glucose, HbA1c concentration, or lipid profile. The subcutaneous fat area correlated significantly with BMI, waist circumference, OGTT-2h-PG, plasma leptin concentration, adiponectin concentration, and CRP concentration. The retroperitoneal fat area correlated better with the systolic and diastolic blood pressure, waist circumference, plasma glycemic indices, lipid profile, CRP concentration, GOT concentration, GPT concentration, and leptin and adiponectin concentrations, as compared to the relationship with subcutaneous fat area. In participants with positive hepatitis B surface antigen or positive hepatitis C antibody (i.e., not hepatitis B carriers and did not have hepatitis C infection), the remaining 267 participants still showed a better correlation between the peritoneal fat area and GOT concentration (peritoneal fat vs. GOT, r = 0.1940, p<0.05; retroperitoneal fat vs. GOT, r = 0.0958, p>0.05 between 2 correlation coefficients) or GPT concentration (peritoneal fat vs. GPT, r = 0.2648, p<0.05; retroperitoneal fat vs. GPT, r = 0.2140, p<0.05 between 2 correlation coefficients).

**Table 3:** Correlation coefficients (r) between body fat and metabolic variables in participants without medications for hypertension, diabetes, or dyslipidemia (N = 353).

	Retroperitoneal fat	p1^a^	p2^b^	Peritoneal fat	p1^a^	p3^c^	Subcutaneous fat	p1^a^	p3^c^
Systolic BP	0.397^a^	<0.001	<0.001	0.350^a^	<0.001	0.07	0.055^b^	0.31	<0.001
Diastolic BP	0.387^a^	<0.001	<0.001	0.325^ab^	<0.001	0.01	0.106^b^	0.05	<0.001
BMI	0.670^a^	<0.001	<0.001	0.645^a^	<0.001	0.14	0.609^a^	<0.001	0.11
WC	0.730^a^	<0.001	<0.001	0.694^ab^	<0.001	0.04	0.542^ab^	<0.001	<0.001
FPG	0.243^a^	<0.001	0.01	0.203^a^	<0.001	0.08	0.067^b^	0.21	0.001
OGTT-2h-PG	0.290^a^	<0.001	0.06	0.279^a^	<0.001	0.67	0.141^ab^	0.008	0.001
HbA1c	0.215^a^	<0.001	0.128	0.202^a^	<0.001	0.65	0.044^b^	0.41	<0.001
Total cholesterol	0.230^a^	<0.001	0.008	0.185^a^	<0.001	0.1	0.074^b^	0.17	0.002
HDL cholesterol	−0.354^a^	<0.001	0.768	−0.399^ab^	<0.001	0.07	−0.082^b^	0.13	<0.001
LDL cholesterol	0.290^a^	<0.001	0.01	0.259^a^	<0.001	0.2	0.084^b^	0.11	<0.001
Triglyceride	0.269^a^	<0.001	0.728	0.299^ab^	<0.001	0.07	0.080^b^	0.13	<0.001
GOT	0.089	0.1	0.03	0.168^ab^	0.002	<0.001	−0.024^b^	0.65	0.03
GPT	0.213^a^	0.001	0.672	0.258^ab^	<0.001	0.03	0.090^b^	0.09	0.04
HOMA2%B	0.206^a^	0.001	0.353	0.215^a^	<0.001	0.74	0.239^a^	<0.001	0.47
HOMA2%S	−0.400^a^	<0.001	0.467	−0.400^a^	<0.001	0.98	−0.360^a^	<0.001	0.44
CRP^d^	0.335^a^	<0.001	0.72	0.376^a^	<0.001	0.14	0.194^ab^	0.07	0.18
Plasma adiponectin^d^	−0.244^a^	<0.001	0.14	−0.237^a^	<0.001	0.755	−0.016^b^	0.05	<0.001
Plasma leptin^d^	0.323^a^	<0.001	0.124	0.327^a^	<0.001	0.97	0.741^ab^	<0.001	<0.001
Plasma renin^d^	0.049	0.21	0.768	0.065	0.06	0.21	0.035	0.45	0.56
Plasma aldosterone^d^	−0.005	0.22	0.01	−0.070^b^	0.03	0.08	−0.012	0.87	0.3

Body fat area was logarithmically transformed for statistical analysis.

BP, blood pressure; BMI, body mass index; WC, waist circumference; FPG, fasting plasma glucose; OGTT-2h-PG, plasma glucose at 2 h during oral glucose tolerance test; HbA1c, hemoglobin A1c; GOT, glutamic oxaloacetic transaminase; GPT, glutamic pyruvic transaminase; HDL, high-density lipoprotein; LDL, low-density lipoprotein; CRP, C-reactive protein.

a p1: retroperitoneal fat, peritoneal fat or subcutaneous fat area vs the indicated metabolic variable.

b p2: retroperitoneal fat vs the indicated metabolic variable, adjusted for peritoneal fat.

c p3: compare with the correlation coefficient between retroperitoneal fat and the indicated metabolic variable.

d log-transformed.

### Retroperitoneal and peritoneal fat areas were associated with incident hypertension

**Figure 3:**
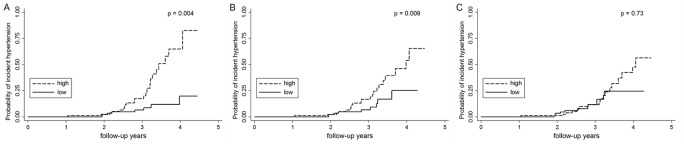
Different fat compartments to predict the probability of incident hypertension. Kaplan-Meier failure curves for the probability of developing hypertension in subgroups divided by the median of (A) retroperitoneal fat area, (B) peritoneal fat area, and (C) subcutaneous fat area. P values by log-rank tests are shown.

After a mean duration of 2.94±0.84 years, 297 (69%) of the 432 participants were followed successfully. Among the 199 participants who did not have hypertension at baseline and have been followed successfully, 32 participants developed incident hypertension. Among the 236 participants who did not have diabetes at baseline, 18 patients developed incident diabetes. Participants with retroperitoneal fat area above the median showed higher risk for incident hypertension, compared with participants with retroperitoneal fat area lower than the median (p = 0.004, Figure 3A). Similar trend can be found for peritoneal fat areas (Figure 3B and 3C), but not for subcutaneous fat areas. In Table 4, retroperitoneal and peritoneal fat areas were associated with the development of incident hypertension in adjusted models, which is different from subcutaneous fat areas (HR for retroperitoneal fat, 1.62 (1.18 2.22), p = 0.003, HR for peritoneal fat, 1.62 (1.12 2.34), p = 0.009, HR for subcutaneous fat, 1.38 (0.90 2.01), p = 0.14). Neither retroperitoneal fat area, peritoneal fat area, nor subcutaneous fat areas was associated with incident diabetes after the adjustment for age, gender and family history of diabetes, which may result from the limited event of incident diabetes in the present study.

**Table 4:** Hazard ratios (HRs) and 95% confidence interval (95% CI) of different fat components to predict the development of incident hypertension and incident diabetes during follow-up.

	Retroperitoneal fat	Peritoneal fat	Subcutaneous fat
Incident hypertension			
Crude (unadjusted)	1.64^a^ (1.25–2.15)	1.77^a^ (1.29–2.41)	1.21 (0.83–1.78)
Model 1	1.62^a^ (1.18–2.22)	1.62^a^ (1.12–2.34)	1.38 (0.90–2.01)
Incident diabetes			
Crude (unadjusted)	1.43^a^ (1.01–2.02)	1.38 (0.91–2.0)	0.72 (0.41–1.2)
Model 2	1.42 (0.89–2.24)	1.46 (0.91–2.35)	0.90 (0.51–1.60)

Hazard ratios were normalized to show the effect of every 1 standard deviation increase in fat areas.

a p<0.05.

Model 1: adjusted for age, sex, and family history of hypertension.

Model 2: adjusted for age, sex, and family history of diabetes.

## Discussion

In the present study, we found that retroperitoneal fat area is associated with MS, plasma adipokines, systemic inflammation, and incident hypertension. Retroperitoneal fat is similar to peritoneal fat, but differs from subcutaneous fat, in its relationship with metabolic syndrome, adipokines, and incident hypertension. Our results indicate that the retroperitoneal fat area should be included as part of visceral fat assessment. Compared with peritoneal fat, retroperitoneal fat is more closely associated with blood pressure and waist circumference, whereas peritoneal fat correlates better with low HDL cholesterol concentration, high triglyceride concentration, and abnormal liver function.

In the literature, there is only one previous study investigating the independent role of retroperitoneal fat to other part of visceral fats in MS [18]. Supporting our findings, the authors of that study also reported a significant association between retroperitoneal fat and MS. However, since all study participants in that study had adrenal diseases (19 had adrenal-dependent Cushing’s syndrome, 12 had subclincal Cushing’s syndrome, and 30 had nonfunctional adrenal incidentaloma), it remained unknown whether the relationship only holds true for participants with adrenal diseases or if the relationship is a consequence of adrenal diseases. In the present study, we included generally healthy participants from the community and confirmed that there is a significant relationship between retroperitoneal fat and MS in the general population.

The mechanisms underlying how visceral adiposity induces metabolic abnormalities are not fully understood. Several hypotheses have been proposed, including increased portal free fatty acid, endocrine function of visceral adipose tissue (adipokines), and visceral obesity as a marker of dysfunctional adipose tissue leading to ectopic fat deposition [4]. For peritoneal and retroperitoneal fat, the blood supply and venous drainage systems are different. Peritoneal fat drains into the portal vein, which may result in increased free fatty acid flux to the liver. High concentration of free fatty acid to the liver leads to the development of fatty liver, insulin resistance, high triglyceride concentrations, and low HDL cholesterol concentrations [19], [20]. Indeed, when epididymal fat pads were transplanted into the mesenterium (portal drainage) or parietal peritoneum (systemic drainage) in mice, only mice with fat transplanted into the mesenterium developed impaired glucose tolerance and hepatic insulin resistance [21]. However, retroperitoneal fat can drain into the renal vein or directly into the inferior vena cava, which may lead to “fatty kidney.” Fatty kidney, as defined by higher renal sinus fat area on CT, has been shown to be associated with hypertension [22]. In addition, there are further differences between different compartments of abdominal fat. Mesenteric pre-adipocytes are found to be distinct from omental pre-adipocytes in their replication potential, mechanism of lipid accumulation, and response to tumor necrosis factor-alpha–induced apoptosis [23]. Retroperitoneal fat has been shown to contain increased amount of brown adipose tissue in humans, as reported by Betz MJ *et* *al* [24]. In mice, Cohen CA *et* *al.* found that intra-abdominal fat depots from different compartments showed distinct patterns in terms of leukocyte to monocyte ratio and gene expression profiles [8]. Indeed, we found different relationships of diastolic blood pressure and plasma HDL cholesterol, triglyceride, GOT, and GPT concentrations with peritoneal and retroperitoneal fat in humans. However, an even greater difference was noted between retroperitoneal fat and subcutaneous fat. The association trends between retroperitoneal fat, metabolic profiles, adipokine concentrations, CRP concentrations, and incident hypertension and diabetes were similar to those of peritoneal fat, but differed from those of subcutaneous fat in the present study.

Taken together, these data suggest that differences exist in the biologic functions of retroperitoneal fat and peritoneal fat. However, compared to subcutaneous fat, retroperitoneal fat and peritoneal fat behave more similarly. Therefore, when the visceral fat area is measured by CT or MRI in human studies for cardiovascular and metabolic diseases, the retroperitoneal fat area should be included.

To the best of our knowledge, the present study is the first comprehensive research to investigate if retroperitoneal fat should be included in the measurement of visceral fat in humans. We analyzed the relationship between retroperitoneal fat, MS, each metabolic abnormality, adipokines, a marker of systemic inflammation, and incident hypertension and diabetes. We also compared the pattern of these relationships among retroperitoneal fat, peritoneal fat, and subcutaneous fat. In addition, the use of CT to measure abdominal adiposity enabled us to compare different compartments of abdominal fat in detail. By contrast, the present study was limited in its follow-up rate of 69%. Although it is not likely that the relationships of retroperitoneal fat to incident hypertension and diabetes will be different in subjects who lost of follow-up, we cannot exclude the possibility completely.

## Conclusion

Retroperitoneal fat is associated with MS, plasma adipokine concentrations, systemic inflammation, and incident hypertension. Retroperitoneal fat is similar to peritoneal fat, but differs from subcutaneous fat, in its relationship with metabolic syndrome, adipokines, and incident hypertension. Our results indicate that the retroperitoneal fat area should be included in the measurement of visceral fat area. In addition, since the relationships between these factors and different compartments of abdominal fat are different, further research is needed to explore the pathophysiologic implications of these findings.

## Supporting Information

Dataset S1 **Cross sectional dataset.**
(XLSX)

Dataset S2 **Longitudinal dataset.**
(XLSX)
